# Lymph Node Ratio: A Novel Prognostic Indicator for Gastric Cancer

**DOI:** 10.7759/cureus.98792

**Published:** 2025-12-09

**Authors:** Anand Praveen Kumar A, Chandralekha K, Sathiyamoorthy P N

**Affiliations:** 1 Medical Oncology, Stanley Medical College, Chennai, IND

**Keywords:** gastric cancer, lymphadenectomy, lymph node ratio, pn stage, prognosis, survival

## Abstract

Introduction

Gastric cancer remains one of the most common malignancies and is a leading cause of cancer-related mortality worldwide. Accurate nodal staging is critical for gastric cancer prognosis but is often compromised by "stage migration" and variability in lymph node (LN) harvest inherent in the current AJCC N-staging system. The lymph node ratio (LNR) has emerged as a potentially more reliable prognostic indicator that accounts for the extent of lymphadenectomy. This study aimed to evaluate the prognostic significance of the LNR, determine an optimal cutoff value, and compare its accuracy against the conventional AJCC 8th edition N-stage in patients undergoing curative gastrectomy.

Methods and materials

This was a single-institution, retrospective cohort study. The study analyzed 85 patients with non-metastatic gastric cancer who underwent surgical treatment between January 2021 and December 2024. Clinicopathological data were collected, and the LNR was calculated and categorized into four groups: LNR1 (0%), LNR2 (1-25%), LNR3 (26-50%), and LNR4 (51-100%).

Results

The median overall survival (OS) was 26 months. Only 24.7% of patients had an optimal LN dissection (>15 nodes). A higher LNR was strongly associated with poorer survival, with median OS decreasing from 36 months in LNR1 to 17 months in LNR4. Multivariate analysis revealed that LNR was a significant independent prognostic factor for survival (p<0.001), whereas the conventional pN stage was not (p=0.098).

Conclusions

The LNR is a simple, robust, and independent prognostic indicator for overall survival in patients with gastric cancer, appearing superior to the conventional pN stage. Its use can improve risk stratification, particularly in cases of suboptimal lymphadenectomy.

## Introduction

Gastric cancer remains a significant global health challenge, ranking as the fifth most common malignancy and the fourth leading cause of cancer-related mortality worldwide [1]. Despite advances in diagnostic and therapeutic strategies, the overall prognosis for patients, particularly those with advanced disease, remains poor. Surgical resection with radical lymphadenectomy stands as the cornerstone of curative treatment for non-metastatic gastric cancer [2]. Accurate postoperative staging is paramount, as it critically informs prognosis, guides adjuvant therapy decisions, and allows for standardized comparison of treatment outcomes across different cohorts.

The current gold standard for staging is the American Joint Committee on Cancer (AJCC) TNM classification system, which relies on the absolute number of metastatic lymph nodes (LNs) to define the pathologic N-stage [3]. However, this method is not without its limitations. A primary concern is the phenomenon of "stage migration," where the accuracy of N-staging is highly dependent on the total number of LNs harvested and examined. A limited lymphadenectomy may result in an underestimation of the true nodal burden, potentially leading to suboptimal treatment for high-risk patients [4]. The variability in the number of retrieved nodes, which can be influenced by the extent of surgery, the diligence of the pathologist, and individual patient anatomy, can therefore compromise the prognostic reliability of the conventional N-staging system [5].

To address these shortcomings, the lymph node ratio (LNR), defined as the ratio of positive LNs to the total number of examined LNs, has emerged as a promising alternative prognostic indicator [6]. The LNR incorporates the total number of nodes examined into its calculation, thereby providing a more standardized and potentially more accurate reflection of the tumor's metastatic potential, minimizing the impact of stage migration associated with an inadequate lymphadenectomy [7]. Several studies across various malignancies have suggested the superiority of LNR over the traditional N-stage, and its utility in gastric cancer has been an area of growing interest [8,9].

Therefore, the objective of this retrospective cohort study was to evaluate the prognostic significance of the LNR in patients who underwent curative gastrectomy for gastric cancer at our institution. We aimed to determine an optimal LNR cutoff value and to compare its prognostic accuracy for predicting overall survival against the conventional N-staging system as defined by the 8th edition of the AJCC manual [3].

## Materials and methods

This is a retrospective cohort study that aimed to investigate the prognostic significance of LNR in gastric cancer patients who underwent surgical treatment. The study included all non-distant metastatic gastric cancer patients who underwent surgical treatment in our Medical Oncology department, Stanley Medical College between January 2021 and December 2024. The data were collected from the medical records in the department.

Inclusion criteria

Histologically confirmed gastric adenocarcinoma, non-distant metastatic disease, and patients who underwent surgical treatment in our department between January 2021 and December 2024 were included.

Exclusion criteria

Patients with metastatic disease, incomplete resection, and missing data were excluded.

Basic demographic data, including age, sex, and other relevant clinical information, were collected from the medical records. The number of LNs dissected and the number of positive LNs were also recorded. The LNR was calculated as the ratio of the number of positive LNs to the total number of LNs dissected.

The LNR was categorized into four groups: LNR1 (0%), LNR2 (1-25%), LNR3 (26-50%), and LNR4 (51-100%). This categorization was based on previous studies that have shown that LNR is a significant prognostic factor in gastric cancer patients.

 The optimal number of LNs dissected was evaluated by dividing the patients into two groups based on the number of LNs dissected, with 15 nodes as the cutoff value. This cutoff value was chosen based on previous studies that have shown that a minimum of 15 LNs should be dissected to ensure accurate staging and prognostication.

Statistical analysis

The statistical analysis was performed using IBM SPSS Statistics for Windows, Version 25 (Released 2017; IBM Corp., Armonk, New York, United States). Descriptive statistics were used to summarize the demographic and clinical characteristics of the patients. Means and standard deviations were calculated for continuous variables, while frequencies and percentages were calculated for categorical variables. Spearman's correlation coefficient (r) was used to examine the correlations among the number of removed LNs, pN stage, and LNR. The Kaplan-Meier survival curve was used to evaluate the survival status of the patients. The log-rank test was used to compare the survival curves among different groups. Univariate analysis was performed using the Cox proportional hazards model to identify significant prognostic factors for overall survival. The hazard ratios (HRs) and corresponding 95% confidence intervals (CIs) were calculated. Multivariate analysis was performed using the Cox proportional hazards model to evaluate the independent prognostic factors for overall survival. The HRs and corresponding 95% CIs were calculated. A two-sided P-value < 0.05 was considered statistically significant.

The following statistical models were used: The Cox proportional hazards model is a statistical model that is commonly used in survival analysis. It is used to model the relationship between the hazard rate and one or more predictor variables. The Kaplan-Meier survival curve is a statistical model that is used to estimate the survival function from lifetime data. It is a non-parametric model that does not require any assumptions about the distribution of the data.

## Results

The patient characteristics showed a median age of 55 years, with a male predominance (71.8%). The majority of patients (61.1%) consumed alcohol, and 52.9% were smokers. Histopathological examination revealed that 49.4% of tumors were poorly differentiated, while 45.9% were moderately differentiated. The tumor site was predominantly distal (80%), and most patients (57.6%) had stage III disease. Only 24.7% of patients had optimal nodes dissected (>15), and the LNR was distributed as follows: LNR1 (0%): 32.9%, LNR2 (1-25%): 15.3%, LNR3 (26-50%): 20%, and LNR4 (51-100%): 31.8% (Table 1).

**Table 1:** Patient characteristics LNR: Lymph node ratio

Patient Characteristics		Frequency
Age	Median age	55 yrs
Sex	Male	61 (71.8%)
	Female	24 (28.2%)
Alcohol	Yes	52 (61.1%)
	No	33 (38.9%)
Smoker	Yes	45 (52.9%)
	No	40 (47.1%)
HPE	Well differentiated	4 (4.7%)
	Moderately differentiated	39 (45.9%)
	Poorly differentiated	42 (49.4%)
Site	Proximal	17 (20%)
	Distal	68 (80%)
Stage	IB	7 (8.2%)
	II	29 (34.1%)
	III	49 (57.6%)
Optimal nodes dissected (>15)	Yes	21 (24.7%)
	No	64 (75.3%)
LNR	LNR1 (0%)	28 (32.9%)
	LNR2 (1-25%)	13 (15.3%)
	LNR3 (26-50%)	17 (20%)
	LNR4 (51-100%)	27 (31.8%)

The correlation analysis revealed a significant positive correlation between the number of removed LNs and pN stage (P < 0.001). This finding suggests that as the number of involve LNs removed, the pN stage also tends to increase. This correlation is likely due to the fact that a higher number of removed LNs increases the likelihood of detecting LN metastases, which in turn affects the pN stage (Table 2).

**Table 2:** Correlation analysis Spearman's correlation coefficient (r) was used to examine the correlations among the total number of removed lymph nodes (LNs), the 8th Edition AJCC pN stage, and the lymph node ratio (LNR). P-values < 0.05 were considered statistically significant

Comparison	Correlation coefficient (r)	P-value	Key Finding / Implication
Correlation Analysis			
Number of removed LNs vs. LNR	0.089	P = 0.432	LNR is not influenced by the extent of surgical lymph node dissection.
Implication of Analysis			
Number of removed LNs and pN stage	0.81	P<0.001	Suggests that as the number of removed LNs increases, the pN stage also tends to increase
Direct Comparison			
pN Stage vs. LNR	0.76	P < 0.001	pN stage and LNR are not interchangeable and provide distinct prognostic information.

In contrast, there was no significant correlation between the number of removed LNs and LNR (P = 0.432). This finding suggests that the LNR is not influenced by the extent of surgical LN dissection. This is an important finding, as it suggests that the LNR may be a more robust and reliable prognostic indicator than pN stage, which can be influenced by the number of removed LNs. Furthermore, a comparison of pN stage and LNR revealed a statistically significant difference between the two (P < 0.001). This finding suggests that the pN stage and LNR are not interchangeable and that they provide distinct prognostic information.

The findings of this correlation analysis have important implications for the management of gastric cancer patients. Firstly, they suggest that the LNR may be a more reliable and robust prognostic indicator than the pN stage, which can be influenced by the number of removed LNs. Secondly, they highlight the importance of considering both the pN stage and LNR when evaluating the prognosis of gastric cancer patients.

**Figure 1:**
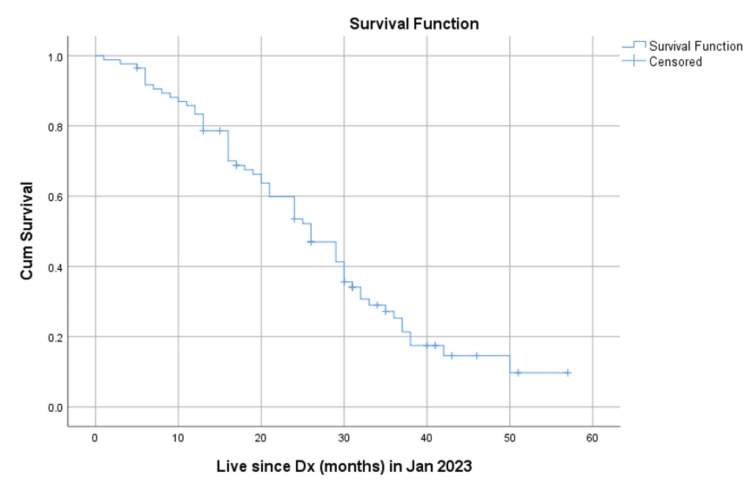
Overall survival Kaplan-Meier survival curves demonstrating overall survival (OS) for patients Median OS- 26 months

**Figure 2:**
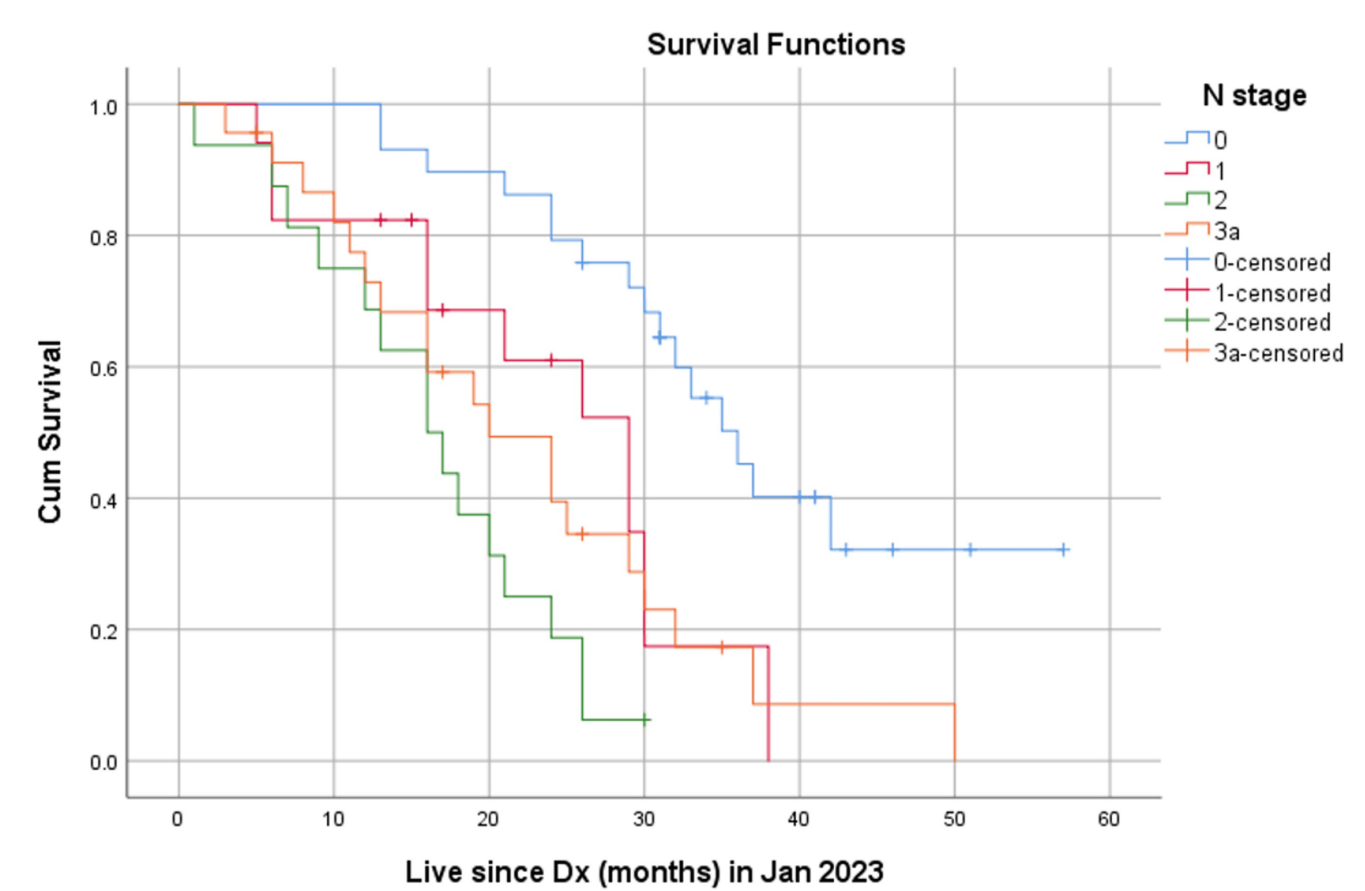
OS based on the pN stage Kaplan-Meier survival curves demonstrating overall survival (OS) for patients stratified by the 8th Edition AJCC pN stage (N0, N1, N2, N3a, N3b). The differences in survival curves among the groups were compared using the log-rank test Median OS: N0-36 months, N1- 29 months, N2- 16 months, N3a- 20 months, p-value< .0001

The median OS for all patients was 26 months (Figure 1). When stratified by nodal status, the median OS was 36 months for patients with N0 disease, 29 months for patients with N1 disease, 16 months for patients with N2 disease, and 20 months for patients with N3a disease (Figure 2).

**Figure 3:**
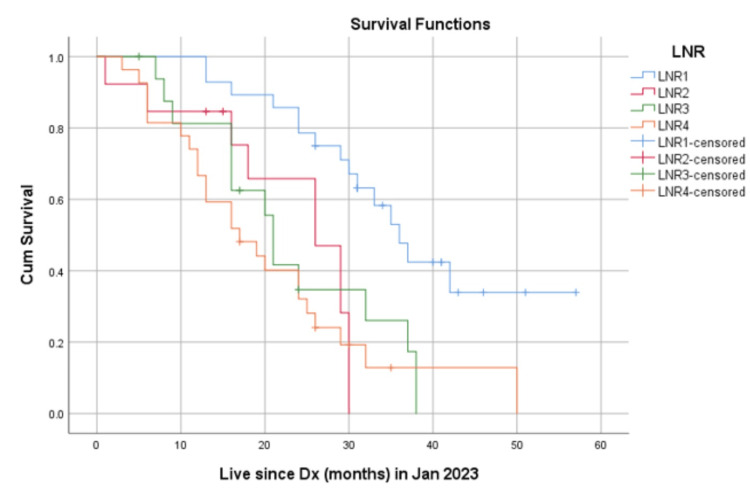
OS based on the LNR Kaplan-Meier survival curves demonstrating overall survival (OS) for patients stratified by lymph node ratio (LNR) categories: LNR1 (0%), LNR2 (1-25%), LNR3 (26-50%), and LNR4 (51-100%). The differences in survival curves among the groups were compared using the log-rank test Median OS: LNR1- 36 months, LNR2- 26 months, LNR3- 21 months, LNR4- 17 months, p-value <.001

When stratified by the LNR, the median OS was 36 months for patients with LNR1 (0%), 26 months for patients with LNR2 (1-25%), 21 months for patients with LNR3 (26-50%), and 17 months for patients with LNR4 (51-100%) (Figure 3). These results suggest that both the nodal status and LNR are significant prognostic factors for overall survival in gastric cancer patients.

The survival curves for nodal status and LNR are shown in Figures 2, 3, respectively. The curves demonstrate a clear separation in survival outcomes based on both nodal status and LNR, with patients having more advanced nodal status and higher LNR values experiencing poorer survival outcomes.

The univariate analysis revealed several significant prognostic factors for overall survival in gastric cancer patients. The HRs and corresponding p-values are presented in Table 3. Histopathological examination (HPE) was not a significant prognostic factor (p = 0.543). However, the T stage and N stage were both significant prognostic factors. Patients with more advanced T stages (T3, T4a, and T4b) had higher HRs, although the differences were not statistically significant (p = 0.539). In contrast, patients with more advanced N stages (N2, N3a, and N3b) had significantly higher HRs, with p-values ranging from 0.007 to <0.001. Optimal LN dissection (>15 nodes) was also a significant prognostic factor, with an HR of 2.086 (p = 0.009). The LNR was a highly significant prognostic factor, with HRs ranging from 3.526 to 3.999 for LNR2, LNR3, and LNR4, respectively (p-values ranging from 0.002 to <0.001).

**Table 3:** Univariate analysis

Parameter		Hazard ratio	P-value
HPE			0.543
	Well differentiated	Ref	
	Moderately differentiated	1.099(0.333-3.623)	0.877
	Poorly differentiated	1.439 (0.438-4.732)	0.549
T Stage			0.539
	T2	Ref	
	T3	0.941(0.496-1.786)	0.852
	T4a	1.074(0.485-2.378)	0.861
	T4b	1.635(0.466-5.732)	0.443
N stage			<0.001
	N1	Ref	
	N2	2.790(1.325-5.875)	0.007
	N3a	6.246(2.916-13.379)	<0.001
	N3b	3.228(1.642-6.343)	0.001
Optimal Nodes		2.086(1.197-3.636)	0.009
LNR			<0.001
	LNR1	Ref	
	LNR2	3.526(1.569-7.920)	0.002
	LNR3	3.038(2.049-6.353)	0.003
	LNR4	3.999(2.049-7.806)	<0.001

A multivariate analysis was performed to evaluate the independent prognostic factors for overall survival in gastric cancer patients. The results are presented in Table 4. The multivariate analysis revealed that optimal LN dissection (>15 nodes) was a significant independent prognostic factor for overall survival (HR = 2.365, 95% CI = 1.221-4.583, P = 0.011). The LNR was also a significant independent prognostic factor for overall survival. Patients with higher LNR values had significantly poorer survival outcomes. The HRs for LNR2, LNR3, and LNR4 were 3.394 (95% CI = 1.502-7.670, P = 0.003), 2.817 (95% CI = 1.338-5.934, P = 0.012), and 4.079 (95% CI = 2.076-8.012, P = 0.009), respectively. In contrast, the N stage was not a significant independent prognostic factor for overall survival (P = 0.098).

**Table 4:** Multivariate analysis

Parameter		Hazard ratio	P-value
N stage			0.098
	N1	Ref	
	N2	2.586(0.287-3.302)	0.397
	N3a	3.689(0.461-9.524)	0.219
	N3b	1.495(0.194-2.529)	0.699
Optimal Nodes		2.365(1.221-4.583)	0.011
LNR			0.001
	LNR1	Ref	
	LNR2	3.394(1.502-7.670)	0.003
	LNR3	2.817(1.338-5.934)	0.012
	LNR4	4.079(2.076-8.012)	0.009

The LNR was a significant prognostic factor for overall survival, with higher LNR values associated with poorer survival outcomes. Multivariate analysis revealed that optimal LN dissection (>15 nodes) and LNR were significant independent prognostic factors for overall survival

## Discussion

In this retrospective cohort study, we evaluated the prognostic significance of the LNR in patients with gastric cancer who underwent curative gastrectomy. Our analysis demonstrates that the LNR is a powerful and independent predictor of overall survival, outperforming the conventional AJCC pN staging system in multivariate analysis. Furthermore, our results underscore the critical importance of achieving an adequate LN yield during surgery.

The principal finding of our study is the independent prognostic value of LNR, which remained significant after adjusting for other clinicopathological factors. This aligns with a growing body of evidence suggesting that LNR is a more reliable prognostic tool than the absolute number of metastatic nodes [5,8]. A meta-analysis involving over 24,000 patients concluded that a high LNR was significantly associated with poorer overall survival, supporting its use as a prognostic marker [8]. Our survival data, which shows a clear dose-response relationship with median OS decreasing from 36 months in the LNR1 group to 17 months in the LNR4 group, reinforces this conclusion.

A key advantage of the LNR highlighted by our results is its independence from the total number of LNs retrieved. We observed a significant positive correlation between the number of examined nodes and the pN stage, but no such correlation with the LNR. This finding directly addresses the well-documented issue of "stage migration" in the TNM system, where an insufficient lymphadenectomy can lead to an artificially lower N-stage and subsequent under-treatment [4,10]. By indexing the number of positive nodes to the total number examined, LNR provides a more standardized measure of the extent of nodal metastasis, irrespective of the surgical or pathological variability in LN retrieval [11]. This stability makes the LNR a particularly valuable tool in cases of suboptimal lymphadenectomy, a significant issue in our cohort, where only 24.7% of patients had more than 15 nodes dissected.

In our multivariate analysis, the LNR emerged as a significant independent prognostic factor, whereas the conventional pN stage did not (p = 0.098). This suggests that the LNR may capture the prognostic information of the pN stage while offering additional predictive power. Similar findings have been reported by other researchers. For instance, a study by Bando et al. also found the LNR to be an independent prognostic factor, particularly in patients with a limited number of retrieved nodes [12]. Our study contributes to the evidence that the LNR is not merely an alternative to pN staging but is potentially superior in its ability to stratify patient risk accurately.

Beyond the LNR, our analysis also confirmed that an optimal LN dissection (>15 nodes) was an independent predictor of improved survival (HR = 2.365, p = 0.011). This is consistent with guidelines from major cancer organizations, which recommend harvesting a minimum of 15 or 16 LNs to ensure accurate staging [2,3]. Achieving this benchmark not only improves staging accuracy but may also have a therapeutic benefit by clearing more potential metastatic foci. The fact that this remained significant in the multivariate model suggests its importance beyond simply enabling better staging.

The findings of this study have important clinical implications. The routine calculation and reporting of the LNR, which can be done at no extra cost, could enhance prognostic accuracy for gastric cancer patients. It could be particularly useful for identifying high-risk patients who might benefit from more aggressive adjuvant therapy, especially in situations where the LN yield is below the recommended standard.

Limitations

We acknowledge several limitations in our study. First, its retrospective nature makes it susceptible to inherent selection bias. Second, being a single-institution study, the results may not be fully generalizable to other populations or healthcare settings with different surgical practices. Third, we did not analyze the impact of adjuvant chemotherapy regimens on survival, which is a potential confounding factor. Future prospective, multi-center studies are needed to validate our findings and to explore how the LNR can be formally integrated into clinical staging systems or treatment algorithms.

## Conclusions

Our study demonstrates that the LNR is a simple, robust, and independent prognostic indicator for overall survival in patients with gastric cancer, superior to the conventional pN staging. Its use can mitigate the confounding effect of variable LN retrieval and improve risk stratification, thereby helping to guide clinical decision-making.
